# Efficient agri-food waste valorization using mealworm (Coleoptera: Tenebrionidae) into nutrient-rich biomass for food and feed

**DOI:** 10.1093/jee/toae035

**Published:** 2024-03-02

**Authors:** John P Musembi, Eunice A Owino, Florence A Oyieke, Chrysantus M Tanga, Dennis Beesigamukama, Sevgan Subramanian, Xavier Cheseto, James P Egonyu

**Affiliations:** International Centre of Insect Physiology and Ecology (icipe), P.O. Box 30772, Nairobi, Kenya; Department of Biloogy, University of Nairobi, P.O. Box 30197, Nairobi, Kenya; Department of Biloogy, University of Nairobi, P.O. Box 30197, Nairobi, Kenya; Department of Biloogy, University of Nairobi, P.O. Box 30197, Nairobi, Kenya; International Centre of Insect Physiology and Ecology (icipe), P.O. Box 30772, Nairobi, Kenya; International Centre of Insect Physiology and Ecology (icipe), P.O. Box 30772, Nairobi, Kenya; International Centre of Insect Physiology and Ecology (icipe), P.O. Box 30772, Nairobi, Kenya; International Centre of Insect Physiology and Ecology (icipe), P.O. Box 30772, Nairobi, Kenya; Faculty of Science and Education, Busitema University, P.O. Box 236, Tororo, Uganda

**Keywords:** agricultural by-product, edible insect, growth performance, bioconversion, nutritional composition

## Abstract

The utilization of yellow mealworm, *Tenebrio molitor* (Linnaeus, Coleoptera: Tenebrionidae), for food and feed is gaining interest globally. However, its production is hindered by expensive commercial diets. This study assessed mealworm growth performance, survival, bioconversion, and nutritional composition when fed on wheat bran (WB) with different inclusion levels (25%, 50%, 75%, and 100%) of Irish potato waste (PW). Results indicated that mealworms fed on diets with 25%–75% PW had increased body length and 1–2 times higher weight gain compared to sole WB and PW diets. The survival rate was 93%–94% across all diets. Mealworms fed on WB had a feed conversion ratio of 3.26, while the efficiency of diet conversion increased with PW inclusion levels. Mealworms fed on diets with 75% PW inclusion had the highest crude fat (48%) and energy levels (598 kcal/100 g), while sole WB produced mealworms with the highest crude protein (55%). The acid detergent fiber achieved using 100% WB was 2- to 3-fold higher, but the crude fiber and neutral detergent fiber did not vary significantly. Considerable amounts of lysine (1.6–2 mg/100 g), methionine (0.5–0.7 mg/100 g), leucine (1.4–2 mg/100 g), and threonine (0.8–1 mg/100 g) were achieved in the mealworm larvae. Our findings revealed that cheap agricultural by-products could be successfully used for the mass production of mealworms, substantially contributing to reduced production costs. Further exploration of the nutrient-dense mealworm larvae for the development of novel food and feed products is crucial.

## Introduction

Insect farming for food and feed is gaining traction as part of the global circular economy to combat increasing food insecurity and malnutrition (Tanga et al. 2021). Insect production is considered a mini-livestock and environmentally sustainable farming strategy as a result of a high feed conversion rate and their capacity to convert low-nutritive wastes into highly nutritious products. Mealworms, for example, have a bioconversion rate of 1.3 billion metric tons per year (Veldkamp et al. 2012) and a feed conversion rate of 3.4–6.1 kilos of ingested feed per kilogram of harvested larvae (Bordiean et al. 2020). Producing mealworms requires less water and land than raising livestock (Oonincx and de Boer 2012, Miglietta et al. 2015). Growing superworm, *Zophobas morio* L. (Coleoptera: Tenebrionidae), or mealworm, *Tenebrio molitor* L. (Coleoptera: Tenebrionidae), requires only 0.2% of all land (Oonincx and de Boer 2012), with only a very small fraction covered by water (Miglietta et al. 2015). In comparison to mealworm production, the total amount of water and land needed to generate 1 g of edible protein in chickens is 2–3 times more, whereas beef production requires up to 14 times the amount of land and 5 times the amount of water (Oonincx and de Boer 2012, Miglietta et al. 2015). Farming mealworms produces fewer greenhouse gases per kilogram of meat compared to pigs and cattle (Premalatha et al. 2011), with similar observations made for beef cattle generating 6–13 times as much carbon dioxide as mealworms do, and broiler birds emitting 32%–167% more carbon dioxide than mealworms (Oonincx and de Boer 2012).

*Tenebrio molitor* is considered a minor secondary pest in flour mills and barns and a contaminant of flour through their cadavers, excretions, and exuviae (Siemianowska et al. 2013). However, it is currently one of the most widely farmed insects for animal feed and human food globally (Benzertiha et al. 2019). Although native to European countries (Ramos-Elorduy et al. 2002), mealworm has spread to several regions around the world, including Africa, Asia, the Mediterranean basin, Australia and Oceania, the United States, Canada, and South and Central America. This species mainly thrives in poorly maintained storage facilities and moldy and damp materials of both plant and animal origin, with the larvae and adults preying on other available insects (Rees 2004). Naturally, *T. molitor* primarily feeds on farinaceous materials like cereals and their products (Ribeiro et al. 2018). However, due to their omnivorous nature, they possess the ability to consume a diverse range of organic materials (Ramos-Elorduy et al. 2002). This remarkable characteristic enables them to play a crucial role in the efficient recycling of organic side streams, ultimately transforming them into valuable, high-quality products. Mealworms are essential in human and animal nutrition (van Huis et al. 2013), provide novel antimicrobial peptides (Chae et al. 2012), and are key in designing the bio-regenerative life support systems used in space missions (Li et al. 2013).

Mealworms are highly enriched with CP and crude fat, ranging from 47% to 60.2% and 19.1% to 36.7%, respectively, with a high level of crude ash, ranging from 2.65% to 6.99% (Hong et al. 2020). The mealworms are rich in amino acids such as lysine, leucine, and valine (Ghosh et al. 2017, Wu et al. 2020). The lysine, methionine, threonine, and tryptophan contents range from 1.58% to 5.76%, 0.52% to 2.2%, 1.57% to 4.29%, and 0.02% to 1.86%, respectively (Hong et al. 2020). Mealworms contain considerable fatty acid contents, such as saturated fatty acids (stearic acid, palmitic acid, and myristic acid) and unsaturated fatty acids, for instance, oleic acid, palmitoleic acid, linoleic acid, linolenic acid, γ-linoleic acid, eicosenoic acid, omega-3 acid, and omega-6 acid (Zielińska et al. 2015, Benzertiha et al. 2020a, Costa et al. 2020, Mattioli et al. 2021). Additionally, mealworms contain considerable levels of vitamins such as B2, B3, B5, B12, E, and H (Finke 2015) and minerals such as magnesium, iron, potassium, zinc, phosphorus, copper, and little calcium (Ghosh et al. 2017, Wu et al. 2020). In recent studies, mealworm commercial farming as a protein feed source for livestock (De Marco et al. 2015, Biasato et al. 2016, Benzertiha et al. 2019, Gasco et al. 2019) and fish (Ng et al. 2001, Barroso et al. 2014, Belforti et al. 2015, Gasco et al. 2016) has become popular around the world, particularly in the United States (Yang et al. 2018), Spain (Reyes et al. 2020), France (Thévenot et al. 2018), and China (Bovera et al. 2015, De Marco et al. 2015, Biasato et al. 2017). In 2020, the larval mealworm market price ranged from USD 10.8–14, 8.4–9.3, 65–70, and 12.9–20 per kg in the United States, China, South Korea, and European Union, respectively, which were higher compared to the price of soybean meal and fish meal, which retailed at USD 0.34 per kg and USD 1.2–1.3 per kg, respectively (Hong et al. 2020).

Mealworms can grow exclusively on WB, which is a popular substrate for their production (Ortiz et al. 2016), with a short developmental time and high weight gain (Bordiean et al. 2022). Vegetable supplementation in the mealworm diet acts as an important source of phytosterols, water, essential fatty acids, and vitamins (van Broekhoven et al. 2015, Ortiz et al. 2016). The vegetables can also enhance the protein content of the diet, thereby shortening mealworm developmental time and increasing their weight gain and survival rate (Morales-Ramos et al. 2011, 2013, van Broekhoven et al. 2015). Morales-Ramos et al. (2013) found that enrichment of WB with dried potato flour significantly shortened the developmental time and improved growth performance, feed utilization, fecundity, and survivorship.

The development of cost-effective diets to supplement the available expensive conventional diets in the production system while enhancing the quality of the produce and yield maximization is essential for mealworm sustainability (Heckmann et al. 2018). In many studies involving mealworm farming, WB emerged as the best diet, but this diet may be scarce in nonwheat-growing countries. Currently, there is no evidence of mealworm farming in East Africa, particularly in Kenya. Considering the scarcity of WB in Kenya and the high cost of its importation from Russia and Ukraine, it is imperative to evaluate alternative locally available agricultural by-products as potential partial or full substitutes for WB in mealworm diets. The mealworms’ ability to feed on various agro-waste provides opportunities for enhanced research efforts toward searching for alternative locally available diets for their mass production in a sustainable way (Harsányi et al. 2020). This study evaluates the impact of substituting WB with agri-food waste, such as PW, on the growth performance, survival, bioconversion, and nutritional composition of an indigenous population of *T. molitor* in Kenya.

## Materials and Methods

### Mealworm Culture

The *T. molitor* mother stock colony was maintained on WB at the Animal Rearing and Quarantine Unit of the International Centre of Insect Physiology and Ecology (*icipe*), Kasarani, Nairobi, Kenya (S 01° 13ʹ14.6″; E 036° 53ʹ 44.5″, 1,612 meters above sea level) following the methods described by Ramos-Elorduy et al. (2002), Ortiz et al. (2016), Morales-Ramos et al. (2013), and Ribeiro et al. (2018) with slight modifications. The eggs collected from the stock colony were transferred into rectangular plastic trays (56 cm × 38 cm × 10 cm) containing 500 g of WB. The WB diet was regularly supplemented with fruits and vegetable cuts to maintain about 70 ± 2% moisture by weight, which was verified with a moisture sensor containing 2 long probes of 12 cm (HydroSenseTM CS620; Campbell Scientific, Inc., Logan, Utah, USA). The growth of the larvae in the culture was observed every day. According to Ortiz et al. (2016), the prepupal stages were chosen from the substrate preserved in other clear rectangular plastic containers (Kenpoly Manufacturer Ltd., Nairobi, Kenya) measuring 18.4 cm × 12.6 cm × 6.7 cm that contained moist wood shavings (sawdust) as pupation substrate. Each container’s lid featured an opening (14.5 cm × 8.3 cm) that was lined with a fine-netting organza material that could hold emerging adult darkling beetles. The rearing conditions were maintained at 28 ± 2.5 °C, 70 ± 2%, and L12:D12 for temperature, relative humidity, and photoperiod regime. The colony has been running for approximately 2 yr (i.e., over 13 generations). The photoperiodic regime was designed based on previous studies (Oonincx et al. 2015, van Broekhoven et al. 2015).

### Diet Preparation

The experimental wheat bran (WB) was purchased from Pembe Flour Mills Limited, Nairobi, Kenya (S 01° 18ʹ26.316″; E036° 52ʹ 25.138″). The Irish potato waste (PW), comprising a mixture of peels and spoiled potatoes, was obtained from Propack Kenya Ltd. Company, Nairobi, Kenya (S 01° 14ʹ44.484″; E 036° 52ʹ 35.147″). The choice of Irish PW was based on its availability in the Kenyan market. The PW substrate was air-dried at *icipes*’ greenhouse at a temperature and relative humidity of 28.5 ± 1.5 °C and 60 ± 2.5% for 1 wk and ground in a grinding mill (Rhino Brand F-35ZS, JB/T6270, Nyagah Mechanical Engineering Limited, Kenya) into fine particles. In each diet (WB and PW), moisture status was assessed in triplicate to standardize the feed formulation, and actual amounts were formulated on a dry matter basis. Briefly, the crucibles were weighed when empty and loaded with fresh sample diets (WB and PW). Samples in the loaded crucibles were dried at 60 °C for 24 h in an oven (WTC Binder FD 115, Tuttlingen, Germany), and the weight of the crucible and dried sample was determined. The dry matter values were averaged. The percentage of dry matter was computed based on Equation (1).


$$\begin{array}{l} \;\%\;\;Feed\;dry\;matter=100-\;\%\;\;Moisture \end{array}$$ (1)

whereby,


$$\begin{array}{l} \;\%\;Moisture=\frac{\left(FSW-WC)-(DSW-WC\right)}{FSW-WC}\times 100 \end{array}$$

WC—cup weight; FSW—fresh sample and cup weight; DSW—weight of the dried sample with cup.

### Experimental Design and Diets Formulation

The experimental design used was a completely randomized design with 5 diet treatments, each replicated 4 times per cycle. The treatments included a diet made of 100% WB (100WB) as a control, 75% WB and 25% PW (75WB/25PW) weight/weight, 50% WB and 50% PW (50WB/50PW), 25% WB and 75% PW (25WB/75PW), and 100% PW (100PW). The diets were subjected to nutritional analysis using the standard methods described, and the results are presented in Table 1.

**Table 1.** Nutritional composition for the test substrates for feeding *Tenebrio molitor*

Nutritional content	WB (control)	75WB/25PW	50WB/50PW	25WB/75PW	100 PW	df	*F*	*P*-value
Dry matter (%)	92.5 ± 0.5a	92.5 ± 0.5a	92.3 ± 0.3a	92.3 ± 0.3a	91.0 ± 0.0a	4, 15	3.15	0.046
Crude protein (%)	15.5 ± 0.1d	14.9 ± 0.1c	13.1 ± 0.2b	12.8 ± 0.1b	12.1 ± 0.1a	4, 15	207.8	<0.001
Crude fat (%)	4.1 ± 0.4b	2.7 ± 0.6ab	2.2 ± 0.0a	3.0 ± 0.3ab	3.8 ± 0.5ab	4, 15	4.05	0.02
Ash (%)	6.2 ± 0.3a	10.5 ± 0.3b	13.3 ± 0.7c	17.8 ± 0.5d	4.4 ± 0.4a	4, 15	132.2	<0.001
Crude fiber (%)	0.35 ± 0.02a	0.39 ± 0.03a	0.40 ± 0.04a	0.41 ± 0.02a	0.42 ± 0.00a	4, 15	2.465	0.0899
Carbohydrates (%)	73.9 ± 0.8b	71.5 ± 0.9b	71.1 ± 0.9b	65.9 ± 0.8a	79.2 ± 1.0c	4, 15	34.7	<0.001
Energy (kcal/100 g)	394.9 ± 1.9d	370.5 ± 2.4c	356.9 ± 2.9b	342.7 ± 0.7a	400.8 ± 1.7d	4, 15	144.8	<0.001

WB, diet made using sole WB; 75WB/25PW, diet made of 75% WB and 25% PW (weight/weight); 50WB/50PW, diet made of 50% WB and 50% PW (weight/weight); 25WB/75PW, diet made of 25% WB and 75% PW (weight/weight); 100 PW, diet made using sole PW. Within each row, means (± SE) followed by the same lowercase letter indicate no significant difference, whereas different lowercase letters within each row indicate larval significant differences in different treatments at *α* = 0.05. *n* = 4.

### Rearing Protocol

A total of twenty thousand 1-day-old larvae were randomly counted from the stock colony and subdivided into 20 groups of 1,000 larvae each. Each treatment replicate contained 1,000 larvae that were reared using the 5 diets described above. In each replicate, a subset of 40 larvae was randomly selected and reared in small containers measuring 184 mm long × 126 mm wide × 67 mm high (Rectangle Food Mate No. 1, Kenpoly Manufacturers Limited) to ensure accurate measurement of larval length weight and survival of *T. molitor* reared on different diets. These containers were placed within the bigger plastic trays (Acme Containers Limited) containing the remaining 960 larvae and measuring 56 cm × 38 cm × 10 cm (length × width × height) to ensure that both groups receive similar conditions (temperature and relative humidity). The 960 larvae were provided with 500 g of the diet, whereas the 40 larvae received a proportionate diet amount of 20.83 g. Fresh green cabbage leaves of 38.4 and 1.6 g were supplied to the 960 and 40 larvae, respectively, on a weekly basis. The fresh cabbage provision was based on a previous study by Kim et al. (2016). The entire experiment was repeated once as a result of the similarity in results obtained during the 2 cycles.

### Data Collection

*Larval growth and survival*. The influence of diet formulations on mealworm growth was determined by measuring the weights and lengths of 40 larvae per treatment at 2-wk intervals from the start of experiments to the maturity stage. The survival computation was also assessed by recording the number of live larvae; dead larvae were regularly removed to minimize the risk of transmitting potential pathogens to live larvae and maintain hygiene (Yang et al. 2018), both in smaller and bigger trays. The experiment was terminated upon the appearance of the first prepupa (63rd day), with most larvae becoming inactive (Bordiean et al. 2020). The final total larval weight was recorded at this stage using a weighing scale.

*Bioconversion performance*. The mealworm bioconversion performance on different diets was evaluated using the efficiency of conversion of ingested feed (ECI) (Equation 2) and the feed conversion ratio (FCR) (Equation 3). This was achieved by first determining larval weight gain and ingested feed weight. The ingested feed weight was determined by subtracting the weight of the residue from the weight of the food provided. The FCR was computed according to Miech et al. (2016), while the ECI was estimated according to Waldbauer (1968). The following equations were used:


$$\begin{array}{l} \begin{array}{lll} & Ef\;f\;iciency\;of\;conversion\;of\;ingested\;feed\;(ECI)\; & =\frac{Final\;larval\;weight\;(g)}{Ingested\;feed\;weight\;(g)}\times 100\; \end{array} \end{array}$$ (2)

$$\begin{array}{l} Feed\;conversion\;ratio\;(FCR)=\frac{Ingested\;feed\;weight\;(g)}{Weight\;gained\;(g)} \end{array}$$ (3)

Where,


$$\begin{array}{ll} Larval\;weight\;gain\;(g)=\; & \;Final\;larval\;weight\;(g)-Initial\;larval\;weight\;(g) \end{array}$$

$$\begin{array}{ll} Ingested\;feed\;weight\;(g)=\; & \;Initial\;feed\;weight\;(g)-Residual\;feed\;weight\;(g) \end{array}$$

### Nutritional Quality of Mealworms Fed on WB-PW Diets

*Sample preparation*. The harvested larvae were starved for 24 h to purge body feces and sacrificed by freezing at −80 °C. The samples were dried in an oven (WTC Binder, FD 115, Tuttlingen, Germany) at 60 °C until a constant weight was achieved, which took approximately 48 h. This was achieved by repeated measurement (12-h intervals) of a sample with a known initial weight until there was on change in weight. The dried larval samples were ground with a laboratory blender (KM–400 mrc) and stored in airtight Ziplock bags. They were preserved in a freezer (a Samsung freezer) maintained at −20 °C until nutritional analysis. The samples were thawed at room temperature, pending laboratory analysis. During nutritional analysis, samples from both experimental cycles were combined per treatment replication. The samples were analyzed for proximate composition (dry matter, carbohydrates, ash, CP, crude fiber, crude fat, energy, NDF, and ADF) and amino acid profile.

*Proximate analysis*. The diets’ and larval crude fat, crude fiber, CP, and ash were determined following the Association of Official Analytical Chemists (AOAC 1990) standard methods. The carbohydrates and energy values were computed as highlighted by Kinyuru et al. (2013) and Duda et al. (2019) in Equations (4) and (5), respectively.


$$\begin{array}{l} \begin{array}{lll} & Carbohydrates=100-fat(\%)-ash(\%)\; & -crude\;protein(\%)-crude\;f\;iber(\%)\; \end{array} \end{array}$$ (4)

$$\begin{array}{l} \begin{array}{llll} & Energy\;value\;(kcal/100\;g)=4\times protein\; & +\;4\times carbohydrate\; & +\;2\times f\;iber+9\times lipid\; \end{array} \end{array}$$ (5)

*Dry matter determination*. In order to calculate the dry matter, AOAC, Method 930.15 was used. Along with the crucible, a 1-g ground sample was weighed and added to the crucible’s weight. The samples were ovendried for 2 h at 135 °C before being desiccated to room temperature. The results were recorded as ovendried weight. The percentage of dry matter was calculated using Equation (6).


$$\begin{array}{l} Dry\;matter\;(\%)=\frac{\begin{array}{c} Oven\;dried\;weight-\;Crucible\;weight \end{array}}{\begin{array}{c} Crucible\;and\;fresh\;sample\;weight-\;Crucible\;weight \end{array}}\times 100 \end{array}$$ (6)

*Ash content determination*. The samples’ ash content was assessed in accordance with AOAC, Method 942.05. This required weighing the dried crucible, adding 1 g of ground sample, and putting them in a muffle furnace (Heraeus-Kundendienst, Dusseldorf, Germany) for 2 h at 550 °C. The temperature of the furnace was changed to 135 °C and allowed to fall. The samples were desiccated for 20 min to allow cooling to room temperature, and the final sample weight was recorded. On a dry matter basis, the percentage ash (dry matter basis) was calculated using Equation (7).


$$\begin{array}{l} Ash\;DM=\frac{Ash\;(\%)}{Fraction\;of\;sample\;DM} \end{array}$$ (7)

Where,


$$\begin{array}{l} Ash\left(\%\right)=\frac{Ashed\;sample\;and\;crubible\;weight-Crucible\;weight}{Fresh\;sample\;and\;crucible\;weight-Crucible\;weight}\times 100 \end{array}$$

*Determination of CP*. Copper catalyst Kjeldahl technique, AOAC, Method 984.13 was used to determine the CP, whereby a 7.5-g mixture of potassium sulfate and copper sulfate at a 9:1 ratio was used as a catalyst together with 15 ml of concentrated sulfuric acid and 1 g of sample, along with blanks. The samples were digested in a DKL 20 Automatic Heating Digester, 32–P 1, with temperature programming ramped at 200 °C for 15 min, 250 °C for 15 min, 350 °C for 30 min, and lastly, 420 °C for an hour. Digested samples were cooled to room temperature and transferred to the distillation and titration system (UDK 159 Automatic Distillation and Titration System, Velp Scientifica, Europe) for the purpose of determining the nitrogen content. As described by Boulos et al. (2020), 5.41 nitrogen-to-protein conversion factor was used (Equation 8).


$$\begin{array}{l} Crude\;protein(\%)=\;\%\;\;N\times F\;(5.41) \end{array}$$ (8)

$$\begin{array}{l} \;\%\;\;Crude\;protein(DM)=\frac{Crude\;protein\;(\%)}{Fraction\;of\;sample\;DM} \end{array}$$ (9)

*Crude fat determination*. In order to extract the fats, the Randall technique was used following AOAC, Method 920.29, whereby 70 ml of the solvent diethyl ether was utilized. This was done using the Soxhlet method in the Soxhlet extractor (Velp Scientifica, SER 148, RS 232, Europe). The extraction cups were dried for 30 min at 105 °C, desiccated for cooling, and weighed. They were then filled with 70 ml of solvent. One-gram samples were placed in extraction thimbles after being tied to filter sheets and weighed. Thirty minutes were spent submerging sample-containing thimbles in a boiling solvent. For a subsequent test sample extraction using a continuous flow of condensed solvent, the samples were washed for 60 min by raising the thimble out of the solvent. Once more, the solvent was recovered after evaporation for 30 min. The fat-filled extraction cups were dried for an additional 30 min at 105 °C to eliminate moisture and solvent remnants. After that, samples were desiccated weighed, and crude fat concentration was determined using Equation (10).


$$\begin{array}{l} \begin{array}{lll} & Crude\;fat\;(\%)\;DM\; & =\frac{Dried\;sample\;and\;crucible\;weight-Dried\;crucible\;weight}{Fresh\;sample\;weight\times (DM\;fraction)}\times 100\; \end{array} \end{array}$$ (10)

*Determination of crude fiber*. AOAC 978.10 was followed in accordance with Weende (2019). The crude fiber was extracted using a fiber analyzer (FIWE Raw Fiber Extractor, Velp Scientifica-Europe) by solubilizing noncellulosic substances using solutions of sulfuric acid and sodium hydroxide. In this instance, a 1-g sample and oven-dried glass crucibles were weighed and placed in a fiber analyzer. Sulfuric acid 1.25% was added to the 150-ml mark, followed by 5 drops of octan-1-ol (antifoam). The samples were boiled for 30 min at 100 °C after being prepared up to the point of boiling. After draining the sulfuric acid, samples were washed 3 times in hot deionized water (30 ml per wash). The procedure above was repeated using a 1.25% solution of sodium hydroxide. Then, the samples were washed 3 times with 30 ml of hot deionized water (100 °C) and once with cold deionized water (20 °C) to cool the crucibles. Thereafter, the samples were washed 3 times with 25 ml of acetone, ovendried at 105 °C for an hour, desiccated, and weighed. This was followed by 3 h of ashing in a muffle furnace at 550 °C and reweighing. The following Equation (11) was used to calculate the percent crude fiber.


$$\begin{array}{ll} Crude\;f\;iber\;(\%)\; & =\frac{\begin{array}{c} Oven\;dried\;sample\;and\;crucible\;weight-\;Ashed\;sample\;and\;crucible\;weight \end{array}}{Fresh\;sample\;and\;oven\;dried\;crucible\;weight}\times 100\; \end{array}$$ (11)

*NDF determination*. This was determined in accordance with Van Soest et al. (1991) and Goering and Van Soest (1970). Ovendried crucibles were weighed; a 1-g sample was recorded, weighed with the crucibles, and fixed in a fiber extractor. Samples were heated until boiling began with the addition of a neutral detergent solution (100 ml), after which they were heated for 1 h at 100 °C, filtered (using pressure and vacuum filter), and washed twice with cold acetone (20 °C) and 3 times with boiling water. The samples were dried for 2 h at 135 °C then cooled in a desiccator before being weighed. They were ashed for 2 h in a muffle at 550 °C, desiccated, and weighed. The calculations were carried out as follows:


$$\begin{array}{ll} NDF(\%)DM\; & =\frac{Oven\;dried\;sample\;and\;crucible\;weight-Crucible\;weight}{\begin{array}{c} (Crucible\;and\;fresh\;sample\;weight-\;Crucible\;weight)\times DM\;fraction \end{array}}\times 100\; \end{array}$$ (12)

$$\begin{array}{ll} Insoluble\;ash\;in\;NDF\; & =\frac{Ashed\;sample\;and\;crucible\;weight-Crucible\;weight}{\begin{array}{c} \left(Fresh\;sample\;and\;crucible\;weight-\;Crucible\;weight)\times DM\;(fraction\right) \end{array}}\times 100\; \end{array}$$ (13)

*Determination of ADF*. ADF determination followed a similar process to that utilized for NDF determination. Instead of neutral detergent solution, 100 ml of acid detergent solution was added to ADF. The ADF was calculated using Equation (14).


$$\begin{array}{ll} ADF\;\;\%\;\;DM\; & =\frac{Oven\;dried\;sample\;and\;crucible\;weight-Crucible\;weight}{\begin{array}{c} (Crucible\;and\;fresh\;sample\;weight-\;Crucible\;weight)\times DM\;fraction \end{array}}\times 100\; \end{array}$$ (14)

*Amino acid profile*. The amino acid profile was determined by weighing 100 mg of each sample into digestion vials. Hydrolysis was done by adding 1.5 ml of 6N HCl, vortexing for 1 min, and digesting samples for 24 h at 110 °C. The hydrolyzed samples were put into Eppendorf tubes, centrifuged at 14,000 rpm for 15 min, filtered, and analyzed by LC-MS.

The LC-MS operating conditions were as follows: a quaternary LC pump (Model 1200) coupled to Agilent MSD 6120-Single quadruple MS with an electrospray source (Palo Alto, CA) was used. The chromatography separation was accomplished on an Agilent system 1100 series (MA, USA) with a ZORBAX SB-C18, 4.6 250 mm, 3.5 µm column, maintained at 40 °C. Water (A) and 0.01% formic acid in acetonitrile (B) were the mobile phases utilized. The gradient employed was 0–8 min, 10% B; 8–14 min, 10%–100% B; 14–19 min, 100% B; 19–21 min, 100%–10% B; and 21–25 min, 10% B. The injection volume was 3 µL, and the flow rate was maintained constant at 0.5 ml/min. At a mass range of *m*/*z* 50–600 and a cone voltage of 30 eV, the mass spectrometer was operated in ESI-positive mode.

Similar LC-MS analyses were performed on serial dilutions of the amino acid standard, which contained 18 amino acids (1–100 ng/µL), Sigma-Aldrich, St. Louis, Missouri, USA), to produce linear calibration curves (peak area vs. concentration) that were utilized for external quantification. Three further amino acid analyses utilizing various batches of samples were conducted.

### Statistical Analysis

For growth, survival, and bioconversion performance, data from experimental cycles 1 and 2 were pooled during statistical analysis, giving a total of 8 replications per treatment. The data were tested for normality using the Shapiro–Wilk test and homogeneity of variance using the Bartlett test. Data that were normally distributed with homogenous variances were subjected to a one-way Analysis of Variance to determine diet effects on mealworm growth and bioconversion performance as well as nutritional quality. The data that did not meet these assumptions was analyzed using the Welch F-test. Survival analysis was done using a generalized linear model fitted with negative binomial distribution. Computation of least squares means was done using “lsmeans” package, followed by mean separation using adjusted Tukey’s method at *P* ≤ 0.05, implemented using “cld” function from the “multicompView” package. The data were analyzed using R software version 4.2.1 for windows (R Core Team 2022).

## Results

### Growth of Mealworm Fed on Mixtures of WB and PW

The inclusion of PW in WB for feeding mealworms significantly affected the length (*F* = 114.1; df = 4, 1487; *P* < 0.001) and weight (*F* = 100.1; df = 4, 35; *P* < 0.001) of the larvae, but not their survival (χ² = 0.031; df = 4; *P* = 0.999) (Table 2). The larvae fed on a WB diet with 25%, 50%, and 75% PW inclusion were approximately 2 mm longer and 1–2 times heavier than those fed solely on WB or PW alone. Meanwhile, poor length and weight performance were observed in mealworms fed solely on PW. The larval survival in all the treatments ranged between 92.5% and 93.8% at the time of harvest.

**Table 2.** Growth performance of *Tenebri*o *molitor* fed on WB with different inclusion levels of Irish potato waste

Parameter	Diet	Time (weeks)				
		2	4	6	8	9
Larval length (mm)	WB (control)	6.35 ± 0.04a	8.48 ± 0.06a	12.59 ± 0.10a	15.08 ± 0.10b	16.29 ± 0.11b
	75WB/25PW	6.32 ± 0.04a	8.77 ± 0.06b	14.30 ± 0.11d	16.68 ± 0.10c	17.81 ± 0.11c
	50WB/50PW	6.37 ± 0.04a	8.95 ± 0.06b	13.55 ± 0.11c	16.83 ± 0.11c	17.62 ± 0.11c
	25WB/75PW	6.45 ± 0.04a	8.84 ± 0.06b	13.08 ± 0.10b	16.71 ± 0.10c	17.66 ± 0.11c
	100 PW	6.45 ± 0.04a	8.92 ± 0.06b	13.21 ± 0.10bc	14.50 ± 0.10a	15.27 ± 0.09a
	df	4, 1595	4, 1521	4, 1501	4, 1491	4, 1487
	*F*	2.453	9.264	37.82	116	114.1
	*P*-value	0.044	<0.001	<0.001	<0.001	<0.001
Larval weight (mg)	WB (control)	1.63 ± 0.07a	3.32 ± 0.12a	11.41 ± 0.45a	23.00 ± 0.59a	27.26 ± 0.52b
	75WB/25PW	1.59 ± 0.08a	3.63 ± 0.11a	19.48 ± 0.46c	37.24 ± 0.34b	40.90 ± 0.37c
	50WB/50PW	1.53 ± 0.07a	3.84 ± 0.15a	20.17 ± 0.44c	35.32 ± 0.42b	39.77 ± 0.79c
	25WB/75PW	1.75 ± 0.07a	3.84 ± 0.16a	21.85 ± 1.05c	35.65 ± 1.12b	38.70 ± 1.39c
	100 PW	1.56 ± 0.10a	3.84 ± 0.16a	16.75 ± 0.25b	22.32 ± 0.55a	22.86 ± 0.67a
	df	4, 35	4, 35	4, 35	4, 35	4, 35
	*F*	1.131	2.716	47.15	123	100.1
	*P*-value	0.358	0.045	<0.001	<0.001	<0.001
Survival (%)	WB (control)	100 ± 0.00a	95 ± 0.04a	94.4 ± 0.04a	93.4 ± 0.04a	93.4 ± 0.04a
	75WB/25PW	100 ± 0.00a	95.6 ± 0.03a	93.4 ± 0.04a	93.4 ± 0.04a	93.1 ± 0.04a
	50WB/50PW	100 ± 0.00a	95.9 ± 0.03a	94.4 ± 0.04a	94.4 ± 0.04a	93.8 ± 0.04a
	25WB/75PW	100 ± 0.00a	95.3 ± 0.03a	94.1 ± 0.04a	93.8 ± 0.04a	93.4 ± 0.04a
	100 PW	100 ± 0.00a	95 ± 0.04a	93.4 ± 0.04a	92.5 ± 0.04a	92.5 ± 0.04a
	df	4	4	4	4	4
	χ²	0	0.022	0.031	0.962	0.031
	*P*-value	1	0.999	0.999	0.916	0.999

WB, diet made using sole WB; 75WB/25PW, diet made of 75% WB and 25% PW (weight/weight); 50WB/50PW, diet made of 50% WB and 50% PW (weight/weight); 25WB/75PW, diet made of 25% WB and 75% PW (weight/weight); 100 PW, diet made using sole PW. Within each column, means (± SE) followed by the same lowercase letter show no significant difference, whereas different lowercase letters within each column indicate larval significant differences in different treatments at *α* = 0.05. *n* = 8.

### Bioconversion Efficiency of *T. molitor* Fed on Various Formulated Diets

The diets’ quality affected *Tenebrio molitor* larval final weight (*F* = 206; df = 4, 35; *P* < 0.001), weight gain (*F* = 207; df = 4, 35; *P* < 0.001), ingested feed weight (*F* = 215; df = 4, 35; *P* < 0.001), efficiency of conversion of ingested (*F* = 84.9; df = 4, 35; *P* < 0.001), and FCR (*F* = 73.5; df = 4, 35; *P* < 0.001) as shown in Table 3. However, the initial larval weight (*F* = 0.4; df = 4, 35; *P* = 0.809) was not significantly different. The final larval weights and weight gain were not significantly different in larvae fed on WB and PW mixtures and were 1–2 times heavier compared to those fed solely on WB and PW. The amount of feed ingested decreased with increasing levels of PW. Apart from the sole PW diet, the efficiency of conversion of ingested feed increased with higher inclusion of PW in the feed, and the reverse was true for the conversion ratio. The larval FCR was 1–2 higher in the larvae fed solely on WB compared to diets with PW.

**Table 3.** Bioconversion performance of *Tenebrio molitor* larvae fed on WB with different inclusion levels of Irish potato wastes

Diets	WB (control)	75WB/25PW	50WB/50PW	25WB/75PW	100 PW	df	*F*	*P*-value
Initial weight (g)	1.37 ± 0.003a	1.36 ± 0.003a	1.37 ± 0.005a	1.37 ± 0.005a	1.37 ± 0.003a	4, 35	0.4	0.809
Final weight (g)	34.35 ± 0.55b	47.40 ± 0.74d	47.43 ± 0.87d	43.96 ± 0.82c	23.46 ± 0.57a	4, 35	206	<0.001
Weight gain (g)	32.98 ± 0.55b	46.04 ± 0.74d	46.06 ± 0.87d	42.59 ± 0.82c	22.10 ± 0.56a	4, 35	207	<0.001
Ingested feed weight (g)	110.6 ± 2.01d	105.5 ± 3.13cd	98.89 ± 0.69c	80.82 ± 1.35b	45.30 ± 0.46a	4, 35	215	<0.001
Feed conversion ratio	3.26 ± 0.10c	2.29 ± 0.07b	2.15 ± 0.05ab	1.90 ± 0.03a	2.06 ± 0.04ab	4, 35	73.5	<0.001
Efficiency of conversion of ingested feed (%)	31.13 ± 0.80a	45.14 ± 1.17b	47.99 ± 1.00bc	54.42 ± 0.88d	51.77 ± 1.04cd	4, 35	84.9	<0.001

WB, diet made using sole WB; 75WB/25PW, diet made of 75% WB and 25% PW (weight/weight); 50WB/50PW, diet made of 50% WB and 50% PW (weight/weight); 25WB/75PW, diet made of 25% WB and 75% PW (weight/weight); 100 PW, diet made using sole PW. Within each row, means (± SE) followed by the same lowercase letter indicate no significant difference, whereas different lowercase letters within each row indicate larval significant differences in different treatments at *α* = 0.05. *n* = 8.

### Proximate Composition of *T. molitor* Larvae Fed on Different Diets

The diet type did not affect larval ash (*F* = 0.976; df = 4, 15; *P* = 0.45), crude fiber (*F* = 1.687; df = 4, 15; *P* = 0.205), or NDF contents (*F* = 2.366; df = 4, 15; *P* = 0.099) (Table 4). However, the mealworms’ dry matter (*F* = 2.625; df = 4, 15; *P* = 0.076), CP (*F* = 34.18; df = 4, 15; *P* < 0.001), crude fat (*F* = 22.56; df = 4, 15; *P* < 0.001), energy content (*F* = 8.322; df = 4, 15; *P* < 0.001), and ADF (*F* = 53.34; df = 4, 15; *P* < 0.001) were significantly affected by the rearing diet recipes. The crude protein (CP) decreased with increasing levels of PW. ADF content was 2–4 times higher in the larvae fed solely on WB. Conversely, the crude fat content of the larvae was comparable for those fed diets with 75% and 100% PW. The energy content was highest in the larvae fed on a diet comprising 75% PW.

**Table 4.** Nutritional composition of *Tenebrio molitor* larvae fed on WB with different inclusion levels of Irish potato waste

Nutritional contents	WB (control)	75WB/25PW	50WB/50PW	25WB/75PW	100 PW	df	*F*	*P*-value
Dry matter (%)	88.8 ± 1.3a	90.3 ± 0.5ab	89.8 ± 0.9ab	90.8 ± 0.9ab	92.5 ± 0.6b	4, 15	2.625	0.076
Crude protein (%)	55.4 ± 1.2c	50.0 ± 0.5b	48.3 ± 0.5b	47.8 ± 0.4b	43.3 ± 0.8a	4, 15	34.18	<0.001
Crude fat (%)	34.9 ± 1.0a	37.4 ± 0.6a	38.4 ± 0.7a	47.7 ± 1.4b	44.6 ± 1.6b	4, 15	22.56	<0.001
Ash (%)	13.8 ± 1.1a	10.8 ± 2.4a	9.7 ± 0.9a	9.9 ± 1.4a	11.0 ± 2.0a	4, 15	0.976	0.450
Crude fiber (%)	0.22 ± 0.00a	0.24 ± 0.03a	0.21 ± 0.01a	0.23 ± 0.00a	0.26 ± 0.02a	4, 15	1.687	0.205
Carbohydrates (%)	0.001 ± 0.0a	1.5 ± 2.2b	3.3 ± 1.3c	0.001 ± 0.0a	0.8 ± 1.2b	4, 15	5.972	0.004
Energy (kcal/100 g)	518.8 ± 7.9a	543.2 ± 11.8ab	552.8 ± 4.4ac	598.2 ± 11.2c	578.3 ± 15.0bc	4, 15	8.322	<0.001
NDF (g/kg)	15.1 ± 0.7a	12.8 ± 0.9a	14.1 ± 0.8a	12.6 ± 0.5a	12.9 ± 0.3a	4, 15	2.366	0.099
ADF (g/kg)	30.8 ± 2.2d	15.9 ± 0.6c	13.8 ± 1.1bc	8.2 ± 0.9a	10.2 ± 0.3ab	4, 15	53.34	<0.001

WB, diet made using sole WB; 75WB/25PW, diet made of 75% WB and 25% PW (weight/weight); 50WB/50PW, diet made of 50% WB and 50% PW (weight/weight); 25WB/75PW, diet made of 25% WB and 75% PW (weight/weight); 100 PW, diet made using sole PW; NDF, neutral detergent fiber; ADF, acid detergent fiber. Within each row, means (± SE) followed by the same lowercase letter indicate no significant difference, whereas different lowercase letters within each row indicate larval significant differences in different treatments at *α* = 0.05. *n* = 4.

### Amino Acid Composition of *T. molitor* Larvae Fed on Different Diets

Of the 14 amino acids measured, 6 amino acids (arginine, histidine, lysine, threonine, glycine, and alanine) were not significantly different across the treatment diets (Table 5). The concentrations of isoleucine (*F* = 3.487; df = 4, 15; *P* = 0.033) and leucine (*F* = 6.623; df = 4, 15; *P* = 0.003) were significantly higher for larvae fed solely on WB. The phenylalanine concentration (*F* = 6.427; df = 4, 15; *P* = 0.003) was significantly higher in larvae fed WB only and/or WB with 25% PW. Glutamic acid was significantly higher in larvae fed WB only and/or WB with 25% PW. Methionine (*F* = 3.249; df = 4, 15; *P* = 0.0.42) and proline (*F* = 4.706; df = 4, 15; *P* = 0.012) concentrations were comparably (but not significantly) higher in the larvae fed 75% WB with 25% PW.

**Table 5.** Amino acids composition of *Tenebrio molitor* fed on WB with different inclusion levels of Irish potato waste

Amino acids (mg/100 g)	Diet					df	*F*	*P*-value
	WB (control)	75WB/25PW	50WB/50PW	25WB/75PW	100 PW			
Arginine^a^	1.88 ± 0.06a	1.91 ± 0.03a	1.86 ± 0.06a	2.06 ± 0.06a	1.90 ± 0.14a	4, 15	0.968	0.454
Histidine^a^	1.28 ± 0.05a	1.37 ± 0.04a	1.36 ± 0.08a	1.42 ± 0.02a	1.26 ± 0.06a	4, 15	1.429	0.272
Isoleucine^a^	1.30 ± 0.04b	1.13 ± 0.02ab	1.02 ± 0.10a	1.14 ± 0.03ab	1.17 ± 0.03ab	4, 15	3.487	0.033
Leucine^a^	2.02 ± 0.08b	1.78 ± 0.04ab	1.38 ± 0.17a	1.63 ± 0.07ab	1.53 ± 0.06a	4, 15	6.623	0.003
Lysine^a^	1.89 ± 0.02a	1.71 ± 0.08a	1.59 ± 0.14a	1.97 ± 0.02a	1.91 ± 0.13a	4, 15	2.84	0.062
Methionine^a^	0.67 ± 0.02ab	0.67 ± 0.01b	0.51 ± 0.07a	0.60 ± 0.04ab	0.63 ± 0.03ab	4, 15	3.249	0.042
Phenylalanine^a^	1.73 ± 0.09b	1.54 ± 0.04ab	1.07 ± 0.19a	1.26 ± 0.09a	1.17 ± 0.06a	4, 15	6.427	0.003
Threonine^a^	0.91 ± 0.02a	0.95 ± 0.01a	0.81 ± 0.08a	0.86 ± 0.03a	0.86 ± 0.03a	4, 15	1.57	0.233
Valine^a^	1.44 ± 0.02c	1.38 ± 0.02bc	1.11 ± 0.11a	1.20 ± 0.04ac	1.17 ± 0.05ab	4, 15	5.985	0.004
Alanine	2.23 ± 0.05a	2.27 ± 0.06a	1.88 ± 0.20a	2.09 ± 0.08a	2.24 ± 0.09a	4, 15	2.181	0.121
Glycine	2.42 ± 0.07b	2.30 ± 0.05ab	1.88 ± 0.23a	2.21 ± 0.10ab	2.18 ± 0.06ab	4, 15	2.748	0.068
Glutamic acid	2.16 ± 0.04b	2.22 ± 0.03b	1.51 ± 0.21a	1.80 ± 0.11ab	1.82 ± 0.09ab	4, 15	6.547	0.003
Proline	1.08 ± 0.02ab	1.13 ± 0.04b	0.89 ± 0.09a	0.94 ± 0.02ab	0.96 ± 0.03ab	4, 15	4.706	0.012
Tyrosine	1.56 ± 0.01c	1.52 ± 0.04c	1.25 ± 0.11ab	1.40 ± 0.06bc	1.08 ± 0.03a	4, 15	10.84	<0.001

WB, diet made using sole WB; 75WB/25PW, diet made of 75% WB and 25% PW (weight/weight); 50WB/50PW, diet made of 50% WB and 50% PW (weight/weight); 25WB/75PW, diet made of 25% WB and 75% PW (weight/weight); 100 PW, diet made using sole PW. Within each row, means followed by the same lowercase letter indicate no significant difference, whereas different lowercase letters within each row indicate larval significant differences in different treatments at *α* = 0.05. *n* = 4.

^a^Essential amino acids.

## Discussion

We evaluated the performance of *T. molitor* on diets comprising various ratios of WB and PW in a trial that was repeated once. We obtained largely consistent results in the 2 sets of trials, which can be attributed to standardization of the protocols and maintaining rearing conditions. We found that *T. molitor* larvae fed on mixtures of WB and PW were 2 mm longer and 1–2 times heavier than those fed on either substrate alone, especially from the fourth week until harvest. It is probable that the mixed diets balanced the nutritional requirements of the larvae (Morales-Ramos et al. 2011, 2013, 2020). The larval weight at harvest in the mixed diets (38.7–40.9 mg per larva) corroborates the results obtained when mealworms were fed on organic vegetable wastes (Harsányi et al. 2020) and attained an average weight of 41 mg. Remarkably higher average larval weights of 140 and 168 mg per larva were reported by van Broekhoven et al. (2015) and Mancini et al. (2019), respectively, when fed using high-protein concentrations in the form of cookies and brewer’s spent grain at a ratio of 1:1. Additionally, high values were also reported by Kim et al. (2016) when mealworms were fed a WB diet supplemented with brewer’s spent grain. These different performances by mealworms on different diets and study locations indicate a need for more research to optimize the rearing of insects under controlled conditions.

The survival of mealworm larvae was high across treatments, ranging from 92.5% to 93.8%, and was not statistically affected by the diet treatments tested. The high survival rate in this study can be attributed to enough food and stocking density per rearing container, thus reducing intraspecific competition for the available food and space. The mealworm’s ability to utilize a range of agricultural organic by-products is also a key driver of the high survival observed in different diets in this study. The survival of *T. molitor* larvae in this study closely corroborates the report by Bordiean et al. (2022) that *T. molitor* larvae survival ranged from 92% to 98% when fed on WB 100%, willowleaf sunflower 25%, and chicken feed 75%. Contrastingly, van Broekhoven et al. (2015) reported a wider range of *T. molitor* larval survival of 71%–91% when fed on commercial diets (Control B-Tm/Za and B-*Ad* from insect rearing companies) and a high-protein/low starch diet (comprising of spent grains, bread remains, beer yeast, and maize distillers’ dried grains with solubles at 30%, 10%, 40%, and 20%, respectively). Several divergent findings on the effect of diet on the survival of mealworms have also been reported. Oonincx et al. (2015) manipulated the protein and fat content of the diet and reported a reduction in survival of 15%–19% on diets that had low protein and high fat, compared to 52%–80% survival on diets that contained high protein and low fat. Mlček et al. (2021) reported *T. molitor* larval survival of 25%, 55%, and 75% on polystyrene foam, WB, and potato, respectively. Silva et al. (2021) reported a survival rate ranging from 66.8% to 81.3% for mealworms fed on poultry litter (comprising of poultry residues and rice husks), substituting the control diet (comprising of barley, milk, chicken feed, oats, and WB at a ratio of 1:1:2:3:3, respectively) with the other 4 poultry litter diets substituted at 25%, 50%, 75%, and 100%. Deruytter and Coudron (2022) reported that weekly frass removal and fresh diets reduced mealworm survival from 97.4% in undisturbed diets to 87%. These divergent findings on the performance of mealworms on WB and other diet compositions in different locations and rearing practices indicate a need for more detailed studies on factors influencing the performance of the insect-based on diet, location, rearing practices, and environmental conditions.

The feed conversion ratio (FCR) values differ depending on diet composition (Scriber and Slansky 1981), insect species, and purpose of the insect (Oonincx et al. 2015). Generally, the higher the diet’s starch compared to protein, the higher the FCR, and vice versa (Bordiean et al. 2020). The lower mealworm FCR value (ranging from 1.9 to 2.29) fed on diet mixtures and solely on PWs meant that the larvae utilized less feed converting to body mass (more efficient feed conversion) compared to the higher FCR (3.26) for larvae fed solely on a WB diet. These results can be corroborated by reported values by Bordiean et al. (2020), whereby mealworm FCR values ranged from 1.57 to 2.08 when fed on chicken feed and WB 100%. However, larvae fed on a willowleaf sunflower diet had an FCR of 4.42. Meanwhile, van Broekhoven et al. (2015) reported an FCR range of 2.62–6.05 based on the diet given and a diverse FCR of 3.8–19.1 observed when mealworms were fed on diets containing different proportions of fats and proteins, with some diets supplemented with carrots.

As in FCR, the efficiency of conversion of ingested feed (ECI) also varies depending on feed quality and insect species. Our findings revealed an ECI value ranging from 31.1% to 54.4%. van Broekhoven et al. (2015) reported a mealworm ECI range of 16.8%–28.9% when fed on bakery remains and other ECI values of 23%–34.4% and 15.8%–33.3% for lesser and giant mealworms, respectively. Compared to other species, Collavo et al. (2005) recorded a higher cricket ECI value of 59% as opposed to Oonincx et al. (2015), whose house cricket had an ECI value ranging from 3% to 9% (control 12%), 16% to 30% for the Argentinean cockroach, and 17% to 24% for the black soldier fly.

The poor performance of *T. molitor* in carbohydrate and energy-denser sole diets could be attributed to the fact that not all sugars are usable by all insects, while some monosaccharides can be toxic because they compete with other essential sugars (Kraus et al. 2019). An optimal level of carbohydrates and energy in *T. molitor* diets, therefore, needs to be determined. The least protein content was recorded in PWs, but this was greatly improved in the mixed diets, with the diet comprising 25% WB and 75% Irish PWs matching the protein content of pure WB. Protein is critical for a wide range of biological functions in insects, such as transport, cell structure, storage, enzymes, and receptor molecules (Kraus et al. 2019). As the Irish PWs were low in protein, future studies on the replacement of WB in the *T. molitor* diet should critically consider alternative protein-rich locally available substrates.

Our analysis of the different diets tested indicated that although they all had comparable levels of crude fiber, crude fat, and dry matter, the mixed diets were richer in ash (the mineral component) and lower in carbohydrates and energy than sole diets. Insects require some minerals, such as coenzymes and metalloenzymes (Chapman 2012). However, CP and ADF were favored by the level of WB, while the converse tended to be true regarding crude fat and energy contents. Generally, mealworm nutritional composition differs depending on the diets’ quality. Its CP ranges from 47% to 60.2%, with an estimated average value of 52.4%, which is higher when compared to conventional soybean meal with a CP of 49.4% (Hong et al. 2020). In our study, the nitrogen-to-protein conversion factor of 5.41 (Boulos et al. 2020) was used, whereby the *T. molitor* fed on a WB diet solely was highly enriched with CP of 55.4%, and the trend decreased in other larvae depending on the amount of substituted WB. This implies that the WB diet had a great influence on mealworm protein composition. The CP range (43.3%–55.4%) achieved in our study conforms to protein values of 44.5% (Bovera et al. 2015), 47.8% (Yoo et al. 2019), 47.7% (Ramos-Elorduy et al. 2002), 46.1% (Ghosh et al. 2017), 46.4% (Ravzanaadii et al. 2012), 60.2% (Heidari-Parsa et al. 2018), 54.8% (Ao et al. 2020), 50.9% (Boulos et al. 2020), and 45.8% (Hussain et al. 2017) while using different organic wastes (WB, Chinese cabbage, cabbage, carrots, radish, and vegetables) for mealworm rearing.

The high values of crude fat (34.9%–47.7%) achieved during the study, especially in *T. molitor* fed on diets containing higher proportions of PW, imply that PW contains higher fat levels compared to WB. These results are higher than the values of fat content of 34.6% (Yoo et al. 2019), 37.7% (Ramos-Elorduy et al. 2002), 34.5% (Ghosh et al. 2017), 32.7% (Ravzanaadii et al. 2012), 31.6% (Ao et al. 2020), 36.1% (Hussain et al. 2017), and 19.1% (Heidari-Parsa et al. 2018) achieved using other substrates. Mealworm ash content (9.73%–13.8%) was higher compared to those reported 6.3% (Yoo et al. 2019), 4.0% (Ghosh et al. 2017), 2.9% (Ravzanaadii et al. 2012), 4.2% (Heidari-Parsa et al. 2018), 3.0% (Ao et al. 2020), and 2.65% (Hussain et al. 2017). The WB diet had the lowest energy value, which is comparable to previously reported values of 554.3 kcal/100 g (Ramos-Elorduy et al. 2002), 539.63 kcal/100 g, and 577.44 kcal/100 g (Rumpold and Schlüter 2013). The higher levels of fat and ash achieved during the study could be attributed to the better nutritional quality of the substrates assessed.

We found that crude fiber was quite lower compared to the values of 6.1% (Yoo et al. 2019), 5% (Ramos-Elorduy et al. 2002), 6.3% (Ghosh et al. 2017), 4.6% (Ravzanaadii et al. 2012), 22.4% (Heidari-Parsa et al. 2018), 4.9% (Ao et al. 2020), and 4.2% (Hussain et al. 2017). The low fiber of *T. molitor* obtained in our study could be attributed to the initially low values in the experimental diets. The highest acid detergent fiber (ADF) recorded in mealworms fed on a sole WB diet is higher compared to the 22.3 g/kg value reported by Finke (2015). In our study, the larval *T. molitor* neutral detergent fiber (NDF) content (12.6%–15.1%) is slightly lower compared to the raw mealworm value (17.4%) reported by Poelaert et al. (2016). Most attention is given to lysine and threonine amino acids detected in larvae that are deficient in commonly utilized cassava, wheat, maize, and rice foods (DeFoliart 1992).

Our findings showed higher lysine and lower threonine contents. Low methionine content was detected across treatments, as reported in previous studies (DeFoliart 1992, Ravzanaadii et al. 2012, Heidari-Parsa et al. 2018), and no cysteine was detected. The presence of arginine in mealworms revealed their benefits for children’s growth, as they are unable to synthesize it in their bodies. The valine, tyrosine, leucine, lysine, alanine, glycine, and glutamic acid levels in our study are lower compared to those reported in several studies (Ravzanaadii et al. 2012, Ghosh et al. 2017, Heidari-Parsa et al. 2018, Ao et al. 2020).

Our findings indicate the high suitability of *T. molitor* produced using the different waste combinations for food and feed purposes. The extended benefits of mealworm meal to livestock and fish have been well documented in the literature. For instance, the growth performance of European sea bass (*Dicentrarchus labrax*) juveniles on a diet with 30% mealworms was compared favorably with a conventional diet (Mastoraki et al. 2020). In another study, the inclusion of mealworms at 5%, 10%, 15%, and 20% in Nile Tilapia juveniles (*Oreochromis niloticus*) diets increased feed intake, specific growth rate, final weight, weight gain, and high FCR compared to the control diet (Tubin et al. 2020).

According to Belforti et al. (2015), 25% and 50% of mealworm inclusion in rainbow trout (*Oncorhynchus mykiss*) conventional diets improved specific growth rate, protein efficiency ratio, and FCR. In Pacific white shrimp (*Litopenaeus vannamei*) (Motte et al. 2019), 50% mealworm inclusion in conventional diets yielded optimum growth. The overall growth and survival of common catfish (*Ameiurus melas*) fingerlings (Roncarati et al. 2015) fed on fish meal (control) and a 50% fish meal diet substituted with mealworms were generally good, with mean weights of 5 and 4 g observed and survival rates of 79% and 70% in the 2 diets, respectively.

In poultry production, mealworm inclusion in small quantities was found to be a better alternative to soybean meal (Hong et al. 2020). The *T. molitor* inclusion in broiler chicks of Ross 708 breed (male) rates of 5%, 10%, and 15% with a control diet formulated based on soy bean meal, corn gluten meal, and corn meal showed a significant increase in body weight (12–25 days old), FCR, and daily feed intake (Biasato et al. 2018). The 0.2% and 0.3% addition of *T. molitor* to broiler chickens (Ross 308 breed females) with a basal diet comprising soybean meal, soybean oil, wheat, fish meal, and rye increased daily feed intake and improved weight gain and FCR (Benzertiha et al. 2020b). Inclusion of *T. molitor* at 0.1%, 0.2%, and 0.3% in broiler chickens showed a decreased feed conversion rate, an increased dressing rate, and weight gain (0–42 days) (Hussain et al. 2017). In the study on barbary partridge (*Alectoris barbara*), 25% inclusion of *T. molitor* on corn-soybean meal (control) showed high live weight at 64 days, and high feed conversion efficiency experienced both at a 25% and 50% inclusion rate (Loponte et al. 2017). The feed conversion efficiency and body weight were significantly improved in Japanese quails (*Coturnix japonica*) when soybean oil and fish meal were substituted with 22.5 and 30 g/kg *T. molitor*, respectively (Zadeh et al. 2019). Contrastingly, some studies showed poor performance when mealworm meal was included in broiler chickens (Biasato et al. 2016, 2017, Bovera et al. 2016). Additional studies to understand the underlining factors that negatively affect poultry production would be crucial.

In human nutrition, mealworm consumption is gaining traction as a result of nutritional composition (Ramos-Elorduy et al. 2002, De Marco et al. 2015, Nowak et al. 2016, Kierończyk et al. 2018), flavor (Jin et al. 2016), digestibility (Yoo et al. 2019), and functional ability (Henry et al. 2015, Song et al. 2018, Benzertiha et al. 2019, 2020), such as antimicrobial peptides and chitin. The current human population growth and protein deficiency increase the demand for potential protein sources to supplement livestock meat production. However, consumer acceptance, reliability, and safety of mealworm consumption dictate its efficacy as a new protein source. Mealworms are globally consumed in different forms, as a whole, in extracts and powders (Aguilar-Miranda et al. 2002, Ghaly and Alkoaik 2009, Zhao et al. 2016). Processing of mealworms after drying and grinding has been reported to reduce them into an easily manageable state and improve marketability as well as consumer acceptability (Aguilar-Miranda et al. 2002). In the food industry, these larvae have been extensively used as appetizers, enriching cookies, bread, cakes, dessert recipes, pie crust, canapes, sui mai tortillas, and burgers (Aguilar-Miranda et al. 2002, Ghaly and Alkoaik 2009).

This study presents a novel potential to utilize and efficiently convert low cost, highly cellulosic industrial waste using native mealworm species. We conclude that PW is an excellent alternative to WB-based diets, yielding comparatively high survival rates for the larvae. It was clearly observed that mixtures of two or more waste types yielded remarkably bigger larvae than solely WB and PW-based diets. However, the CP, most amino acids, and ADF contents, as well as the FCR of the larvae, were significantly influenced by the diet types. Our study demonstrated that readily accessible agri-food waste may be utilized to successfully raise high-quality yellow mealworm larvae. One might infer that the optimum diets for mealworm growth were those that contained 75% and 100% WB based on the tested properties, such as FCR.

This study supplements, for the first time, the list of organic waste substrates that can be potentially used in mealworm farming by producers as a result of their availability and cost-effectiveness. Furthermore, this work is in line with the current Green Environment policy, which seeks to promote a circular economy and short supply chains by applying sustainable production and consumption, supporting the utilization of renewable materials, and reducing toxic pollutants and waste. However, it is required to adapt diverse combinations of biowastes as feed materials in an insect’s diet in order to facilitate the bioconversion of nutrients from various organic waste streams by mealworms. Future studies to optimize substrate combinations are crucial to allow researchers and mealworm producers to obtain more reliable results in terms of optimal growth, low FCR, higher efficiency of the conversion of ingested diets, optimal composition of biowaste, and high nutrient-rich larvae for human food and animal feed.
